# Interfacial Forces in Free-Standing Layers of Melted Polyethylene, from Critical to Nanoscopic Thicknesses

**DOI:** 10.3390/polym14183865

**Published:** 2022-09-15

**Authors:** Fernando Iguazú Ramírez-Zavaleta, Victor Manuel Torres-Dominguez, Gonzalo Viramontes-Gamboa, José Luis Rivera

**Affiliations:** Facultad de Ciencias Físico—Matemáticas, Universidad Michoacana de San Nicolás de Hidalgo, Morelia 58000, Mexico

**Keywords:** polyethylene, free-standing layers, vapor/liquid equilibria, molecular dynamics, surface tension, pressure, thermal stability

## Abstract

Molecular dynamics simulations of ultrathin free-standing layers made of melted (373.15–673.15 K) polyethylene chains, which exhibit a lower melting temperature (compared to the bulk value), were carried out to investigate the dominant pressure forces that shape the conformation of chains at the interfacial and bulk liquid regions. We investigated layer thicknesses, $t_{L}$, from the critical limit of mechanical stability up to lengths of tens of nm and found a normal distribution of bonds dominated by slightly stretched chains across the entire layer, even at large temperatures. In the bulk region, the contribution of bond vibrations to pressure was one order of magnitude larger than the contributions from interchain interactions, which changed from cohesive to noncohesive at larger temperatures just at a transition temperature that was found to be close to the experimentally derived onset temperature for thermal stability. The interchain interactions produced noncohesive interfacial regions at all temperatures in both directions (normal and lateral to the surface layer). Predictions for the value of the surface tension, γ, were consistent with experimental results and were independent of $t_{L}$. However, the real interfacial thickness—measured from the outermost part of the interface up to the point where γ reached its maximum value—was found to be dependent on $t_{L}$, located at a distance of 62 Å from the Gibbs dividing surface in the largest layer studied (1568 chains or 313,600 bins); this was ~4 times the length of the interfacial thickness measured in the density profiles.

## 1. Introduction

Interfacial forces are responsible for several phenomena in polymeric systems [1,2,3]. Multicomponent polymer blends develop into either single-phase envelopes, multi-faceted envelopes, or completely phase-separated structures depending on the ability of its components to blend with and spread on one another; their spreading coefficients are dependent on the surface tension, γ, of the blend components as well as the polar and dispersive components of γ [4]. Agents that increase the compatibility of blend components accomplish this by reducing the interfacial tension between the polymer phases and stabilizing the phase morphologies [5,6,7,8]. Linear polyethylene (HDPE) forms compatible blend systems that have low interfacial tensions with block copolymers such as styrene−ethylene−butylene−styrene (SEBS) and styrene−ethylene−butylene (SEB). Furthermore, blend systems that are incompatible with polystyrene can become compatible when SEB and SEBS are used as agents at the interface [9].

Polymeric [10,11] and atomistic [12] ultrathin layers become mechanically unstable at thicknesses below a critical thickness, $t_{c}$, breaking into droplets or developing mechanically stable holes with diameters of a few nm. A previous study [11] on $t_{c}$ of unsupported fluid layers of melted polyethylene chains found that the thickness of the layers did not affect the interfacial thickness even when the layer thicknesses is as low as $t_{c}$. Unsupported fluid layers that approach ultrathin thicknesses at $t_{c}$ are the result of processes such as drying, stretching, or are under other external forces or conditions that usually eliminate interfacial material. It has been proposed that capillary waves induce the corrugation of the layer, causing the interfaces to become extremely close, interacting with each other and resulting in the development of disjoining forces that induce film breakage [13,14,15,16]. However, simulations conducted on films at their $t_{c}$ did not identify capillary waves large enough to corrugate unsupported layers of melted polyethylene chains, and still they break at thicknesses below $t_{c}$ [11]. In general, the cohesivity of a system formed of ultrathin layers is different from that of the bulk material, which leads to changes in other properties of the film, such as the glass transition temperature, $T_{g}$ [17,18,19,20], and the melting temperature, $T_{m}$ [21,22].

In this paper, we investigated the local interfacial pressures (normal and lateral) and their contributions (kinetic, intrachain, and interchain) in the interfacial and the bulk liquid regions. We also related the local pressure profiles obtained from these investigations to the dominant conformations (such as stretched and extended chains, as well as cohesive and noncohesive regions) of free-standing layers comprised of melted polyethylene chains in a vacuum at a range of thicknesses from the limit of mechanical stability to lengths of tens of nm. Total pressure profiles were also used to predict the nature of the surface tension and the true interfacial length, which was found to extend several nm into the “bulk liquid region” as indicated by the interfacial density profiles. Future work will investigate variables such as the effects of the size of the simulation cell on the properties calculated and data dispersion, the effects of agents aimed at increasing the compatibility of the interfaces, and the interfacial forces of polymer blends that are in contact.

## 2. Methods

Simulations of free-standing layers of melted polyethylene chains with macroscopic areas cannot be conducted at the atomic level due to clear computational limits. However, the study of samples with small areas at the nanoscopic scale under periodic conditions are considered to be reliable systems that can predict the behavior of thermophysical and interfacial properties (with large data dispersion) of systems with macroscopic areas [11,12,23]. The properties of free-standing layers can be considered to be at vapor/liquid equilibrium (VLE) when they are under specific environmental conditions or at vacuum–liquid equilibrium. Molecular dynamics (MD) simulations are commonly employed to calculate phase equilibriums and the interfacial properties of atomic and molecular systems, including polymeric systems [24,25,26]. In this study, we employed MD simulations using the Large-scale Atomic/Molecular Massively Parallel Simulator (LAMMPS) software [27]. Simulations of the periodic systems were confined within a periodic simulation cell in the shape of a rectangular parallelepiped with two square faces. The surface layer was developed on the square face of the parallelepiped located at the center of the simulation cell, which had an interfacial area of $A_{i}=145\;Å\times 145\;Å$. The square sides were large enough to contain a stretched C_200_ chain in each lateral direction. Previous simulations have demonstrated that the designated interfacial area is large enough to correctly predict the liquid density of melted polyethylene chains [11], but simulations conducted on model atomic systems show that much larger areas are required to significantly reduce the errors in predictions of interfacial properties [12,23]. We found either vacuum or melted liquid and interfacial regions along the axis perpendicular to the free-standing surface (the inhomogeneous direction); positions within this axis were denoted as *z*. The initial conformation of the chains formed a layer that fully covers one face of the simulation cell, it developed into the free-standing film, and the periodic conditions did not let the system change the face where the free-standing layer initially developed. The periodicity along the normal direction results in a central layer which periodically repeats along the normal axis, but the size of the simulation cell in this direction resulted in a separation between the layers large enough to avoid the interactions between the sites of the central layer and the repeating layers in the normal direction.

We conducted simulations using layers of C_200_ polyethylene chains because Dee and Sauer [28] found a dependence between the size of the chain and their interfacial properties, which narrowed at larger chain sizes. The difference between C_150_ and C_2000_ chains was relatively small; thus, the properties of C_200_ chains are expected to be similar to those of polymeric systems with thousands of monomeric units. Simulations with much larger chain sizes will require the use of larger interfacial areas, which will, in turn, require prohibitive computational resources. Layers of chains at temperatures between 373.15 and 673.15 K were simulated using the NVT ensemble, where the number of particles and volume of the simulation cell were held constant, while the temperature fluctuated around a constant value, which employed the Nosé thermostat and a timestep of 1 fs [29]. Melted states are expected in the range of temperatures studied because they are greater than $T_{m}$, which reduces to *T* < 373.15 K in ultrathin polyethylene layers [21,22]. In contrast, $T_{m}$ has a value of 403.65 K in bulk states for chains with a molecular weight of 100.4 kg/mol [22]. However, the range of studied temperatures is lower than the limit of thermal stability (5% weight loss is observed at 695.55 K, while the maximum loss occurs at 747.45 K) [30,31]. The initial layers had conformations that consisted of ordered and stretched chains that were parallel to the interfacial area; they were equilibrated in periods of 1 ns for systems at temperatures above 573.15 K and 3 ns for layers at temperatures below 573.15 K, with the equilibration criteria being the equilibration of the interfacial forces. In a previous paper, we calculated density profiles using the equilibration of the average bulk liquid density as the equilibration criteria, and all systems equilibrated in periods on the scale of hundreds of ps [11].

Intrachain and interchain interactions were computed using the Transferable potential for phase equilibria (TraPPE) forcefield [32], and the united atom potential was calculated using the Lennard–Jones (LJ) potential for interchain interactions and intrachain interactions separated by more than four bonds. Coarse-grained forcefields have been used widely to describe structural properties of bulk phases of polymers and under phase equilibrium [33,34], but the features resolution of the pressure profiles are in the range of a few Angstroms [35,36], which are not possible to describe with coarse-grained forcefields because the size of the beads are sometimes larger than the required resolution. Bond (b) and angle vibrations were computed using harmonic potentials, while dihedral vibrations were calculated using a cosine function [37]. Interchain interactions were calculated using a large cutoff radius, $r_{c}=7.5\;\sigma_{CH_{3}-CH_{3}}$, where $\sigma_{CH_{3}-CH_{3}}$ is a LJ parameter for the end group CH_3_. This radius was large enough to fully account for all significant interactions, reproduced experimental interfacial properties, and avoided the use of the long-range corrections both during and after the simulations in systems that are fully described with LJ potentials [36,38] or even ionic systems [39,40]. Calculations using the TraPPE potential using C_50_ chains and C_192_ chains have reproduced experimental bulk values for $T_{m}$ [41] and $T_{g}$ [42], respectively.

Total pressure profiles and their contributions (kinetic, LJ, as well as bond, angle, and dihedral vibrations) are distributed locally in small slabs along the normal direction. The profiles were calculated using Harasima contours [43], a feature available in LAMMPS [44], which arbitrarily distributes the stress from two interacting sites between the two originating slabs. While there is no unique definition for the local pressure, Harasima contours correctly describe interfacial pressures as the Irwing–Kirkwood contours [45] in planar interfaces; however, they are problematic for curved interfaces [46,47]. From the total pressure profiles, we calculated γ for the equilibrated layers using their mechanical definition [43,45]:

(1) $$\gamma =\frac{1}{2}\int_{-\infty }^{\infty }\left[P_{⊥,T}\left(z\right)-\langle P_{∥,T}\left(z\right)\rangle \right]dz,$$

where $P_{⊥,T}\left(z\right)$ and $P_{∥,T}\left(z\right)$ are the total pressure profiles in the directions that are normal and lateral to the surface layer. $P_{∥,T}\left(z\right)$ is averaged using the values from the two lateral directions. Equation (1) considers the existence of two interfaces.

The calculation of the pressure profiles suffers from dispersion problems, with spontaneous displacements of whole layers along the normal direction during the simulations. This issue can be addressed by displacing the entire system every time the center of mass of the system is displaced by more than a specified distance (1 Å), while problems with layer breathing [48,49] were addressed by using one of the interfaces, the Gibbs Dividing’ surface, $z_{GDS}$, as a reference position, and shifting the position of entire instantaneous profiles (calculated every 100 timesteps) to match their $z_{GDS}$. The reference position was obtained by fitting the density profiles to the following expression for liquid phases surrounded by vacuum:

(2) $$\rho \left(z\right)=\frac{1}{2}\rho_{l}-\frac{1}{2}\rho_{l}\;tanh\left(\frac{z-z_{GDS}}{d}\right),$$

where $\rho_{l}$ is the average bulk density. The “10–90” interfacial thickness, $t_{i}=2.1790d$ [25,36], is the region over which the density increases from 10% ($z_{10\%})$ to 90% ($z_{90\%})$ of $\rho_{l}$. Layer thicknesses, $t_{L}$, were measured as the average separation between the location of the two $z_{10\%}$.

## 3. Results

**Figure 1:**
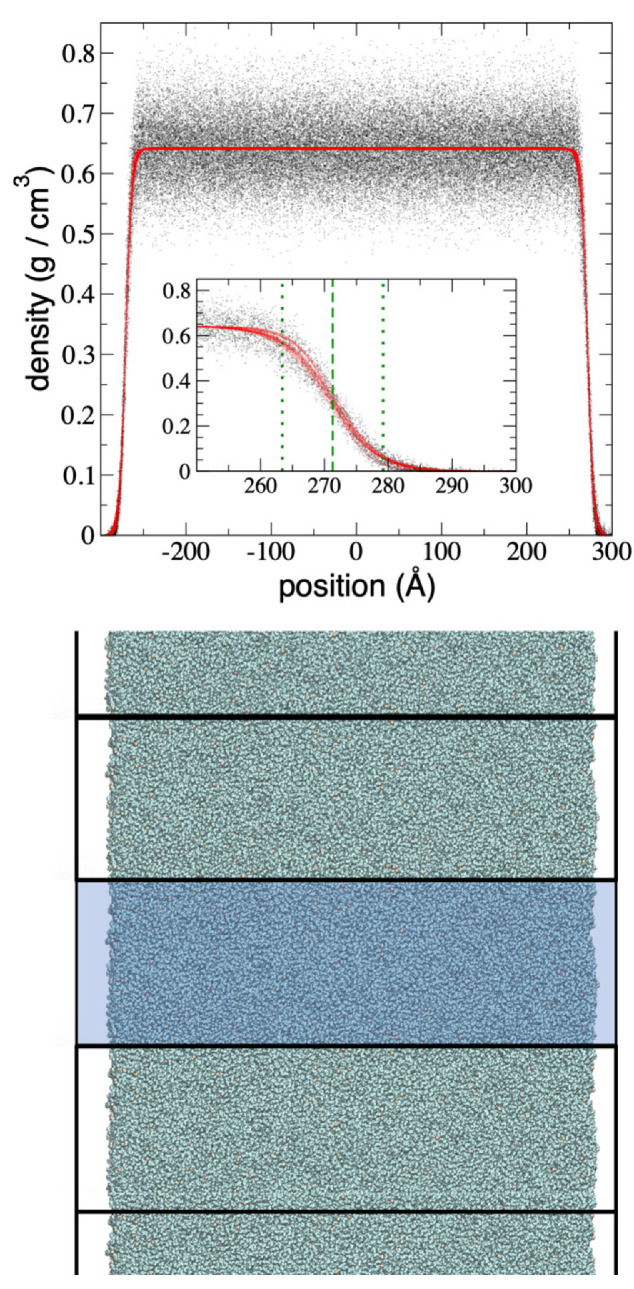
(**Top**) Density profile of a free-standing layer of melted polyethylene chains as a function of their inhomogeneous position within the simulation cell at 673.15 K (black dots). Red dots represent the fits to Equation (2). Inset: An expanded view of the right interfacial region between 250 and 300 Å with the same axes and units as the original plot. The dashed green line corresponds to the position of the $z_{Gds}$, while the dotted green lines correspond to the positions of $z_{10\%}$ (**right**) and $z_{90\%}$ (**left**). (**Bottom**) Snapshot of the central simulation cell (highlighted in blue) and the central cell replications in one of the lateral directions.

MD simulations of free-standing fluid layers comprised of melted polyethylene chains were performed at layer thicknesses ranging from $t_{c}$ to nanoscopic lengths. In a previous paper, the $t_{c}$ was estimated as a function of the temperature by removing interfacial chains of a constant interfacial area until the layers became mechanically unstable, causing part of the surface layer to retract, leaving holes in the form of pores with diameters of a few nm [11]. Using an $A_{i}$ as described in the present work, it was predicted that 94 polyethylene chains are needed at 373.15 K ($t_{c}$ = 3.42 nm and $t_{i}$ = 0.82 nm), while 116 are needed at 673.15 K ($t_{c}$ = 5.63 nm and $t_{i}$ = 1.59 nm), the limit of mechanical stability. We simulated systems with an increasing number of chains, up to 1568 (313,600 LJ sites); this produced a layer thickness of 43.36 and 55.85 nm at 373.15 and 673.15 K, respectively, while the values of $t_{i}$ were unaffected across the entire range of thicknesses studied [11]. We calculated density profiles at intervals of 100 steps and obtained the $z_{GDS}$ for each profile, which were later used to correct the pressure profiles. In the top of Figure 1, the density profile of the polyethylene layer with 1568 chains at 673.15 K is shown along with the parameters that characterize the interface. The density profile is plotted along the normal direction to the interfacial surface and describes the changes in population of the united atoms along this direction within the central simulation cell (Figure 1, bottom). Replicates in the two lateral directions of the central simulation cell are used to compute the interactions of united atoms in the central simulation cell with united atoms in the replicated simulation cells to simulate a free-standing layer with infinite interfacial surfaces. Compared to the points that made the density profile, the fits to Equation (2) are very uniform, while the simulation points in the density profiles exhibited large dispersion due to the small $A_{i}$ used.

### 3.1. Total Pressure Profiles

**Figure 2:**
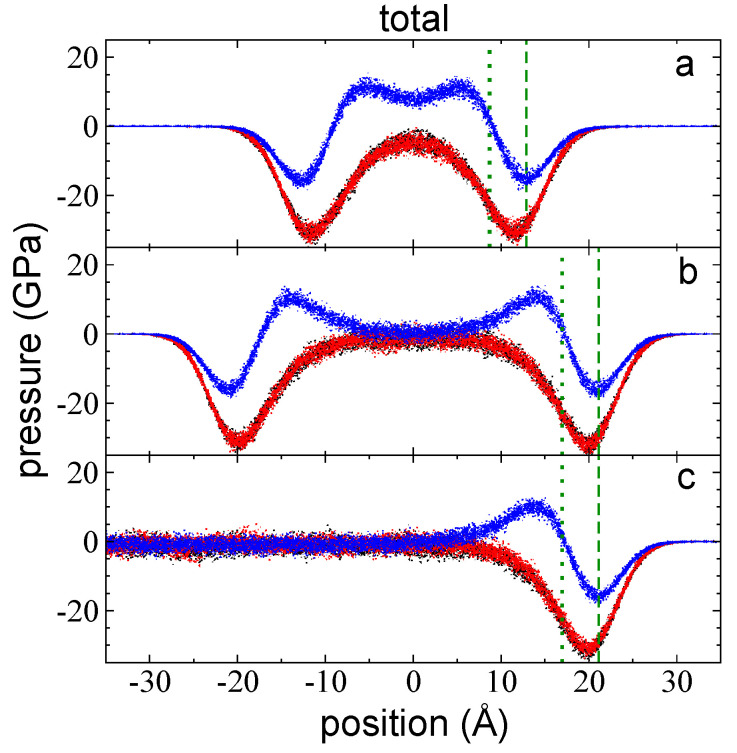
$P_{⊥,T}$ (blue points) and $P_{∥,T}$ profiles (black and red points) as a function of their inhomogeneous position within the simulation cell for layers of melted polyethylene chains (C_200_) with thicknesses of (**a**) 94 chains (the $t_{c}$), (**b**) 155 chains, and (**c**) 288 chains. In this diagram, $A_{i}=$ 145 Å × 145 Å and *T* = 373.15 K. The dotted and dashed green lines represent the positions of $z_{GdS}$ and $z_{90\%}$, respectively.

The total pressure profiles for flexible chains of polyethylene are the sum of the contributions due to interchain and intrachain interactions plus kinetic contributions. Pressure profiles were computed to study the interfacial cohesive forces that maintain mechanical stability of ultrathin layers at their $t_{c}$. In Figure 2a–c, the $P_{⊥,T}$ and average $P_{∥,T}$ profiles for 3 thin layers of melted polyethylene chains at *T =* 373.15 K are shown as a function of their position on the normal axis to the interfacial surface at a layer thickness that corresponds to the $t_{c}$ (94 chains, Figure 2a), as well as for wider layers containing 155 (Figure 2b) and 288 chains (Figure 2c). To observe the effects of layer thickness on the interfaces, we shifted the profiles of the layer with 288 chains in the normal direction to match the $z_{GdS}$ of the layer with 155 chains. The profiles at its $t_{c}$ are similar to those obtained for the free-standing thin layers of LJ atoms [12,23]. In each polyethylene layer, when starting from the vacuum at either interface (right or left) and moving towards the center of the liquid layer, we found an initial negative peak, which indicates that the interfaces are dominated by cohesive forces in all directions. The magnitude of the peaks was the same in the lateral directions and lower in the normal direction, and this magnitude remained constant regardless of the layer thickness. The position of the negative peaks in the $P_{⊥,T}$ profiles always appeared first (as one moves towards the center of the layer), suggesting that they are the outermost cohesive region of the layer. The minimum in the negative $P_{∥,T}$ peaks appeared later and are ~2 Å away from the minima observed in the negative $P_{⊥,T}$ peaks; this difference in positions could be the cause of layers with small areas and curved surfaces develop when larger flat layers break. The negative $P_{∥,T}$ peaks also indicate the presence of a cohesive region but in the lateral directions. Interestingly, the positions of all the negative peaks in the $P_{⊥,T}$ profiles match their corresponding position relative to the $z_{Gds}$; in other words, the highest cohesivity in $P_{⊥,T}$ is obtained when the density at the interface is half the value of its bulk liquid density. For layers with thicknesses equal to their $t_{c}$, the $z_{Gds}$ is the position at which the density is half the value of the density at the center of the layer. For $P_{∥,T}$, the minimum is reached when densities are slightly higher than half the bulk liquid density.

The interfacial region in the pressure profiles extended well inside the bulk liquid region: for the widest layer, isotropic pressures, which characterize a bulk phase, are reached at distances of ~14 Å from $z_{90\%}$, while the distance between $z_{90\%}$ and $z_{Gds}$ is ~4 Å at 373.15 K. Towards the center of the layer (Figure 2), pressure profiles in both directions exhibited different behaviors. For profiles where $t_{L}$ > $t_{c}$, the magnitude of the $P_{∥,T}$ profiles decreased, exhibiting a small, negative plateau as the interfacial region is reached. Beyond the interfacial region, at the center of the layer, where the bulk liquid regions develop, the magnitude of $P_{∥,T}$ fluctuated around a constant value. A larger bulk liquid region developed in the layers with greater layer thicknesses, while $t_{i}$ remained constant for all layers, including the layer where $t_{L}$ = $t_{c}$. In the layer where $t_{L}$ = $t_{c}$, the magnitude of $P_{∥,T}$ also decreased in the interfacial region but a bulk liquid region did not develop; this resulted in a profile that looked like a combination of two interfacial regions. In atomic systems, bulk liquid regions only developed in layers where $t_{L}\;\geq$ 1.6 nm [12,23]; bulk liquid regions did not develop in layers with smaller $t_{L}$. In contrast, the polyethylene melts studied in this work developed bulk liquid regions at $t_{L}$ ≥ 6.0 nm. However, we did not perform refined simulations to determine the minimum $t_{L}$ required to develop a bulk liquid region in either system.

Unlike the $P_{∥,T}$ profile, the $P_{⊥,T}$ profiles (Figure 2) displayed positive peaks after the outermost negative peaks. The positive peaks represent noncohesive zones that are slightly smaller than the outermost cohesive zones (negative peaks). They are likely the result of the interactions between chains in the bulk liquid region (or at the center of the layer in samples where $t_{L}$ = $t_{c}$) and chains in the outermost cohesive zone, which compress the chains between them, resulting in the development of the noncohesive zone. The magnitude of the positive peaks decreased as $t_{L}$ increased, from ~14 GPa ($t_{c}$, 94 chains) to ~12 GPa (288 chains); this may be associated with the size of the bulk liquid regions, which are wider and most likely showed more anisotropic pressures in thicker layers. In the thicker layer (288 chains), beyond the positive peak (towards the center of the layer), the magnitude of $P_{⊥,T}$ decreased in the interfacial zone, plateauing as the interfacial zone ended. In the bulk liquid region, the $P_{⊥,T}$ profile fluctuates around the same small negative value (−1.6 GPa) observed in the corresponding $P_{∥,T}$ profile. Negative pressures arise at nanoscale layer thicknesses due to the prevalence of disjoining forces, which become more important as $t_{L}$ decreases; in contrast, capillary forces dominate in the micro- and macro-scale layers [50]. Negative pressures exhibited in thin layers have been reported for confined fluid layers [51,52] as well as the free surfaces of non-evaporating supported layers [50,51]. The layer with an intermediate $t_{L}$ (155 chains) exhibited behavior that was similar to that observed in the thickest layer, but the plateau values reached in the $P_{⊥,T}$ profile are slightly larger than those observed in the corresponding $P_{∥,T}$ profile, which suggests that this layer is still too thin to be considered a system at VLE. The similarly of values in both pressure profiles can be used as a criterion to determine if a system is in real VLE or it represents a metastable state. In the layer where $t_{L}$ = $t_{c}$, the $P_{⊥,T}$ profile slightly decreased but never approached values similar those observed in the corresponding $P_{⊥,T}$ profile; the entire profile once again looked like a fusion of the two interfaces. In the profiles obtained in atomic systems [12,23], the magnitude of the $P_{⊥,T}$ profile never decreased at the center of the layer, which is likely due to the small size of the atomic systems (0.34 nm for Ar atoms) compared to the volume of the polyethylene chain, which can take several conformations, from expanded coils to globular states, which are expressed as a distribution of values between 1 and 2.8 nm with an expected value of 1.6 nm at 373.15 K [11].

The overall picture of the total profiles reveals an outermost region that pulls any chains that are somehow freed from the interface in the normal direction and into the vacuum. In contrast, the base of those chains, which are located at the interface, are at separations large enough to produce attractive forces, while the base of those chains attracts each other in the lateral directions.

### 3.2. Contributions to the Total Pressure Profiles

**Figure 3:**
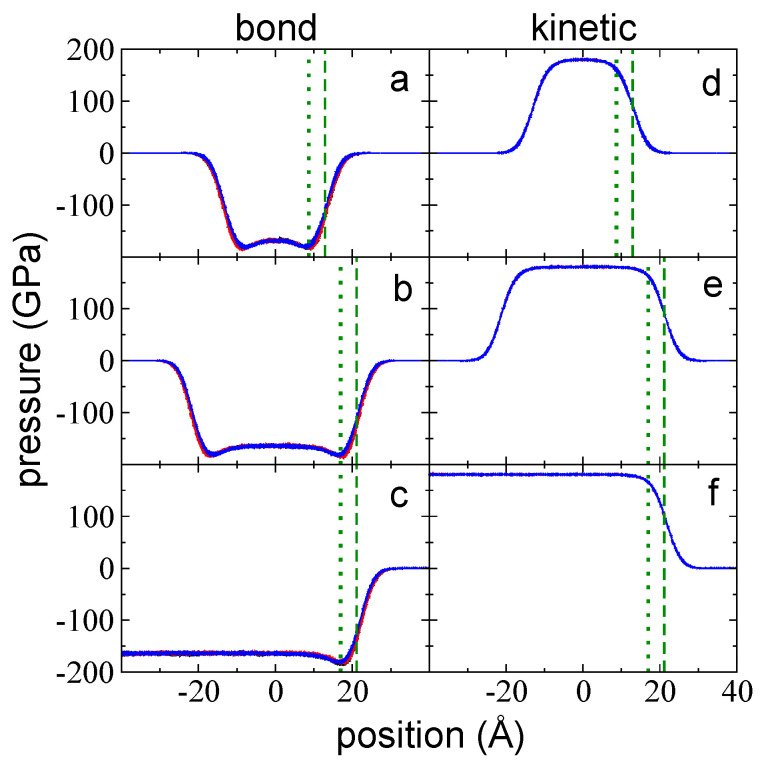
The $P_{⊥,b}$ (blue points) and $P_{∥,b}$ profiles (black and red points) as a function of the inhomogeneous position within the simulation cell for layers of melted polyethylene chains (C_200_) with thicknesses of (**a**) 94 chains (the $t_{c}$), (**b**) 155 chains, and (**c**) 288 chains. In this diagram, $A_{i}=$ 145 Å × 145 Å and *T* = 373.15 K. (**d**–**f**) The corresponding $P_{⊥,k}$ (blue points) and $P_{∥,k}$ (black and red points) profiles. The dotted and dashed green lines represent the positions of $z_{GdS}$ and $z_{90\%}$, respectively.

Using Harasima contours [43], we calculated and analyzed all contributions distributed in the inhomogeneous direction of the pressure profiles. These contributions include intrachain interactions (bond, angle, and dihedral vibrations), interchain interactions (LJ interchain, including 1–5 intrachain), and kinetic contributions. Unexpectedly, the largest contributions to the total pressure profiles were bond vibrations and kinetic contributions rather than interchain interactions. The bond vibrations and kinetic contributions were of the same order of magnitude at the center of the layers at 373.15 K (kinetic contributions were always positive and slightly larger, while bond vibration contributions were always negative). In contrast, the interchain interactions were one order of magnitude lower than the kinetic or bond interactions. Net negative peaks due to intrachain interactions (bond, angle, and dihedrals) were the result of regions in which the sum of forces due to stretched conformations of chains were larger than the sum of forces due to compressed conformations; in positive peaks, compressed conformations dominate the sum of forces. Pressure profiles due to bond vibrations in both directions ($P_{⊥,b}$ and $P_{∥,b}$) for all the layers studied at 373.15 K are shown in Figure 3a–c. Each profile exhibits negative values, indicating that the stretched conformations dominate the distribution of conformations in both the interfacial and the bulk liquid regions. Moving from the vacuum to the center of the layers, $P_{⊥,b}$ and $P_{∥,b}$ grew in the interfacial region, reaching a minimum peak at a position that was close to the position of $z_{90\%}$, which is very close to the limit of the bulk liquid zone. Beyond this point, the magnitude of both profiles decreased, plateauing at the limit of the bulk liquid zone.

Kinetic contributions ($P_{⊥,k}$ and $P_{∥,k}$) can only make positive contributions to the pressure tensor (Figure 3d–f). These profiles look like the symmetrical complement of the bond vibration profiles without the minimum peaks; similar to the bond vibration profiles, they are very uniform, and no appreciable differences were observed between $P_{⊥,k}$ and $P_{∥,k}$, which is likely due the scale used to plot the profiles. Towards the center of the layer, the kinetic contributions grew in the interfacial region, plateauing at the limits of the bulk liquid zone.

**Figure 4:**
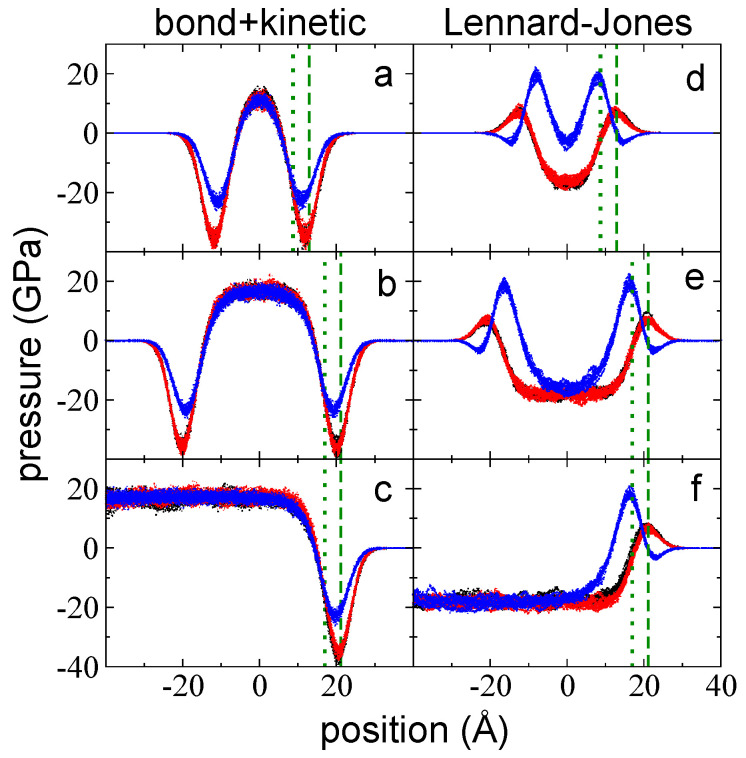
$P_{⊥,b+k}$ (blue points) and $P_{∥,b+k}$ profiles (black and red points) as a function of the inhomogeneous position within the simulation cell for layers of melted polyethylene chains (C_200_) with thicknesses of (**a**) 94 chains (the $t_{c}$), (**b**) 155 chains, and (**c**) 288 chains. $A_{i}=$ 145 Å × 145 Å and *T* = 373.15 K. (**d**–**f**) The corresponding $P_{⊥,LJ}$ (blue points) and $P_{∥,LJ}$ (black and red points) profiles. The dotted and dashed green lines represent the positions of $z_{GdS}$ and $z_{90\%}$, respectively.

The sum of the bond vibrations and kinetic pressure profiles, the main contributions to the total pressure profiles, reveal the differences between $P_{⊥,b+k}$ and $P_{∥,b+k}$ at the interfacial region (Figure 4a–c). The summed profiles exhibited minimum peaks in both directions; for $P_{∥,b+k}$, the peaks are located at the $z_{GdS}$, while for $P_{⊥,b+k}$, they are located between $z_{GdS}$ and $z_{90\%}$, and are lower than the peaks of $P_{∥,b+k}$. These profiles exhibited interfacial regions that extended well beyond the position of $z_{90\%}$ in the widest layer, which was ~16 Å beyond $z_{90\%}$. In the summed profiles, the bulk liquid zone at the center of the layer exhibited similar positive values in both directions that were one order of magnitude larger than those observed in the total pressure profiles. This suggests that kinetic pressures can not only cancel contributions due to bond vibrations but can also cancel contributions from the remaining interactions at 373.15 K.

Interchain pressure profiles ($P_{⊥,LJ}$ and $P_{∥,LJ}$) accounted for pressures due to LJ interactions, which not only include between sites in different chains but also due to sites in the same chain that are separated by five or more bonds. Net negative peaks in these pressure profiles reflect regions where the sum of forces due to attractive interactions are larger than the sum of repulsive interactions; conversely, repulsive interactions dominate at positive pressure peaks. Repulsive (positive values) and attractive (negative values) contributions are due to interactions between sites at separations that are shorter and larger than the LJ parameter σ, respectively, where $\sigma_{CH_{3}-CH_{3}}=3.95\;Å$, $\sigma_{CH_{2}-CH_{2}}=3.75\;Å$, and $\sigma_{CH_{3}-CH_{2}}=3.85\;Å$ represent the separations between two LJ sites at which the interaction force between the sites is zero. Pressure profiles for $P_{⊥,LJ}$ and $P_{∥,LJ}$ are shown in Figure 4d–f for the three studied layers at 373.15 K. At the interfacial region, only repulsive peaks developed, with very small attractive peaks in $P_{⊥,LJ}$ found at the outermost region of the interface. The interfacial region for these profiles also developed deeper into the bulk liquid regions, ~16 Å beyond $z_{90\%}$. Interestingly, the location of the positive peaks corresponded to $z_{90\%}$ ($P_{⊥,LJ}$) and to $z_{GdS}$ ($P_{∥,LJ}$), because $z_{GdS}$ and $z_{90\%}$ are two positions geometrically defined in the density profiles. The maximum in $P_{⊥,LJ}$ most likely requires interfacial regions that are very close to the “bulk liquid region” defined in the density profile but retain some flexibility to interact with the outermost part of the interface in an anisotropic way. The maximum in $P_{∥,LJ}$ most likely requires some spacing between the chains in the lateral directions to exist to maximize the pressures in these directions, which can develop in regions where the density is lower than the bulk liquid region. However, why the maximum is present exactly at regions where the density is half the bulk liquid region needs to be clarified in future studies. The contributions due to angle and dihedral vibrations are insignificant when compared to the rest of the contributions; thus, the total pressure profiles are produced by summing the LJ and the bond vibrations and kinetic profiles. In the normal direction, $P_{⊥,T}$ retained the positive peak in $P_{⊥,LJ}$ and the negative peaks in $P_{⊥,b+k}$ because they are at different positions in the inhomogeneous direction. However, the positive peak in $P_{∥,LJ}$ did not survive because it is in the same position as the peak in $P_{∥,b+k}$, which is larger than $P_{∥,LJ}$. The negative values in the total pressure profiles at the bulk liquid region developed because the negative contributions derived from intrachain, and interchain interactions are greater than the kinetic contributions.

**Figure 5:**
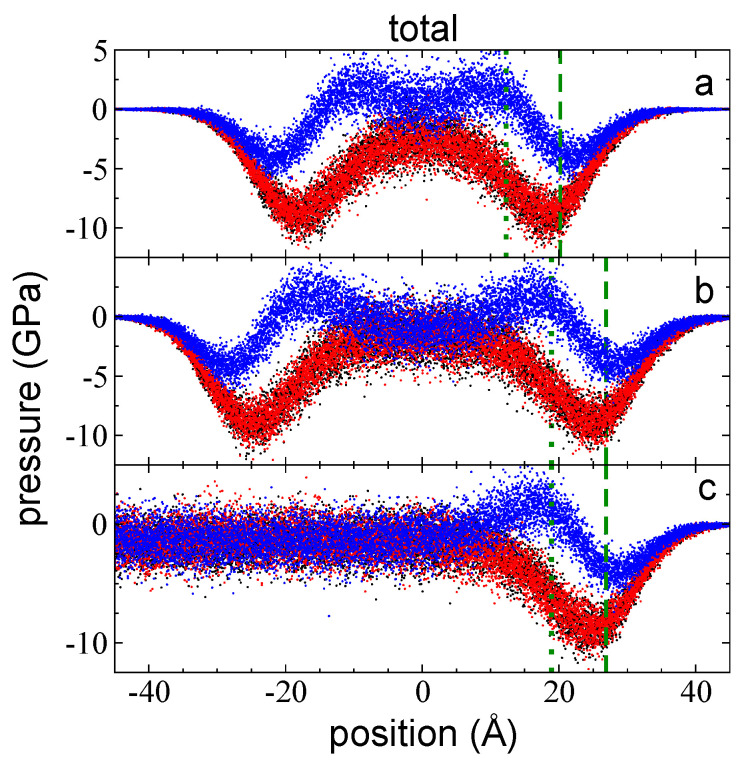
$P_{⊥,T}$ (blue points) and $P_{∥,T}$ profiles (black and red points) as a function of the inhomogeneous position within the simulation cell for layers of melted polyethylene chains (C_200_) with thicknesses of (**a**) 94 chains (the $t_{c}$), (**b**) 155 chains, and (**c**) 288 chains. $A_{i}=$ 145 Å × 145 Å and *T* = 673.15 K. The dotted and dashed green lines represent the positions of $z_{GdS}$ and $z_{90\%}$, respectively.

At higher temperatures (*T* = 673.15 K), $P_{∥,T}$ and $P_{⊥,T}$ profiles had the same positive and negative peaks as observed in the interfacial regions of the profiles produced at 373.15 K (Figure 5a–c); however, the magnitude of these peaks is considerably lower. The lower interfacial pressures produce smaller differences between $P_{⊥,T}$ and $P_{∥,T}$, resulting in a smaller γ at this temperature. At this temperature, 116 chains are needed to maintain the mechanical stability of the layer where $t_{L}$ = $t_{c}$. The position of the minima in the negative peaks of $P_{⊥,T}$ in all layers corresponded to $z_{GDS}$, similar to the observations in the profiles at 373.15 K. The magnitudes of the positive and negative interfacial peaks did not change with $t_{L}$. At the center of the layer, pressures developed increasingly isotropic distributions as $t_{L}$ increased. The layer where $t_{L}$ = $t_{c}$ exhibited anisotropy in $P_{∥,T}$ and $P_{⊥,T}$, resembling the behavior of the layer with an intermediate $t_{L}$ (155 chains) at 373.15 K, and indicating that the anisotropy between $P_{∥,T}$ and $P_{⊥,T}$ in layers at their $t_{C}$ are primarily due to the length of $t_{L}$.

**Figure 6:**
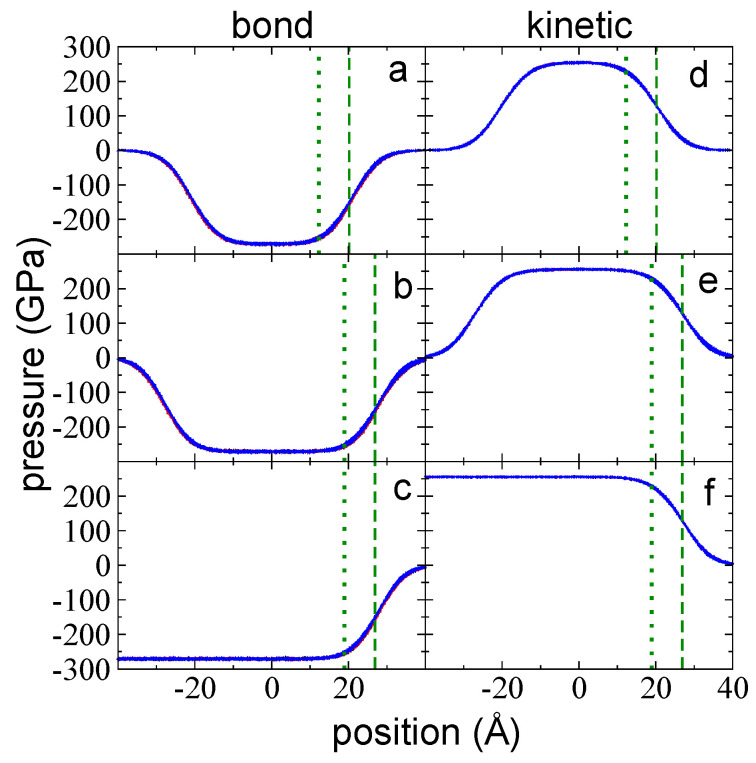
$P_{⊥,b}$ (blue points) and $P_{∥,b}$ profiles (black and red points) as a function of the inhomogeneous position within the simulation cell for layers of melted polyethylene chains (C_200_) with thicknesses of (**a**) 94 chains (the $t_{c}$), (**b**) 155 chains, and (**c**) 288 chains. $A_{i}=$ 145 Å × 145 Å and *T* = 673.15 K. (**d**–**f**) The corresponding $P_{⊥,k}$ (blue points) and $P_{∥,k}$ (black and red points) profiles. The dotted and dashed green lines represent the positions of $z_{GdS}$ and $z_{90\%}$, respectively.

At 673.15 K, kinetics and bond vibrations are still the largest contributors to the total pressure (Figure 6). Once again, these two contributions had values with opposite signs and, in general, they exhibited the same behavior as the profiles at 373.15 K, except that the interfacial regions in $P_{∥,b}$ and $P_{⊥,b}$ did not develop minimum peaks. In general, the profiles grew to asymptotic values at the center of the layers. The pressures grew monotonically at the interfaces of all layers, reaching intermediate values at $z_{GDS}$ and plateauing very close to $z_{90\%}$. At the center of the layers (the bulk liquid regions), the isotropic pressures of $P_{∥,b}$ and $P_{⊥,b}$ were larger than that of $P_{∥,k}$ and $P_{⊥,k}$, contrary to the trends observed at 373.15 K, indicating a possible transition temperature that will be analyzed in conjunction with the LJ interactions (interchain plus 1–5 intrachain).

**Figure 7:**
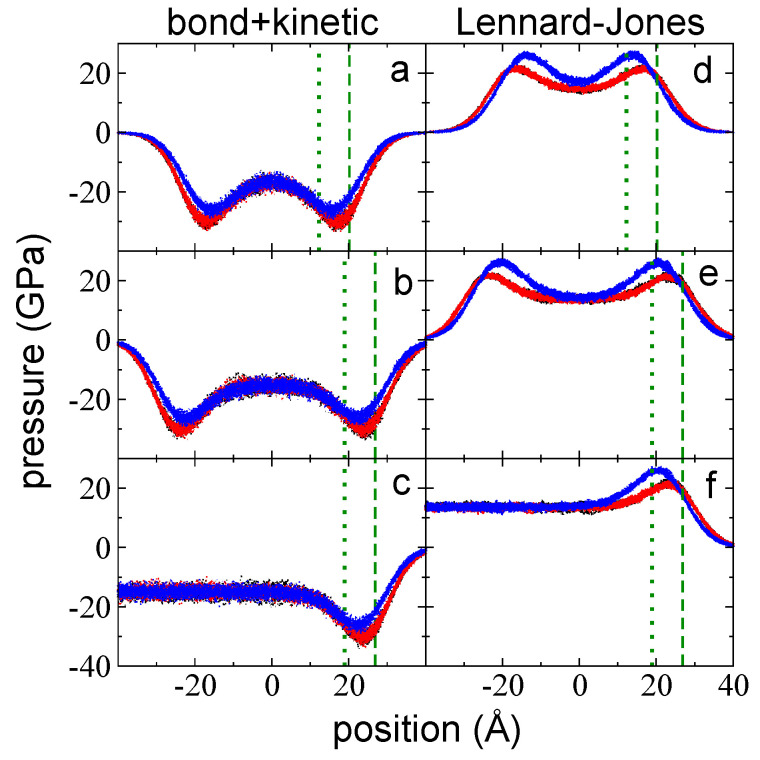
$P_{⊥,b+k}$ (blue points) and $P_{∥,b+k}$ profiles (black and red points) as a function of the inhomogeneous position within the simulation cell for layers of melted polyethylene chains (C_200_) with thicknesses of (**a**) 94 chains (the $t_{c}$), (**b**) 155 chains, and (**c**) 288 chains. $A_{i}=$ 145 Å × 145 Å and *T* = 673.15 K. (**d**–**f**) The corresponding $P_{⊥,LJ}$ (blue points) and $P_{∥,LJ}$ (black and red points) profiles. The dotted and dashed green lines represent the positions of $z_{GdS}$ and $z_{90\%}$, respectively.

The sum of the contributions of kinetics and bond vibrations at 673.15 K produced profiles with only negative values (Figure 7a–c). Similar to the behavior of the profiles at 373.15 K, $P_{∥,b+k}$ and $P_{⊥,b+k}$ exhibited slightly different profiles at the interfacial regions, with negative peaks between $z_{GDS}$ and $z_{90\%}$. Unlike the profiles at 373.15 K, the profiles did not grow to positive values at the center of the layers (the bulk liquid regions); instead, they slightly increased and plateaued at a negative value. The LJ pressure profiles (Figure 7d–f) exhibited positive peaks at the interfacial regions similar to those observed in the profiles at 373.15 K, but they did not decrease until reaching negative values at the center of the layers; instead, they remained positive, in contrast to the profiles at 373.15 K, where they reached negative values.

### 3.3. Limit of Thermal Stability

**Figure 8:**
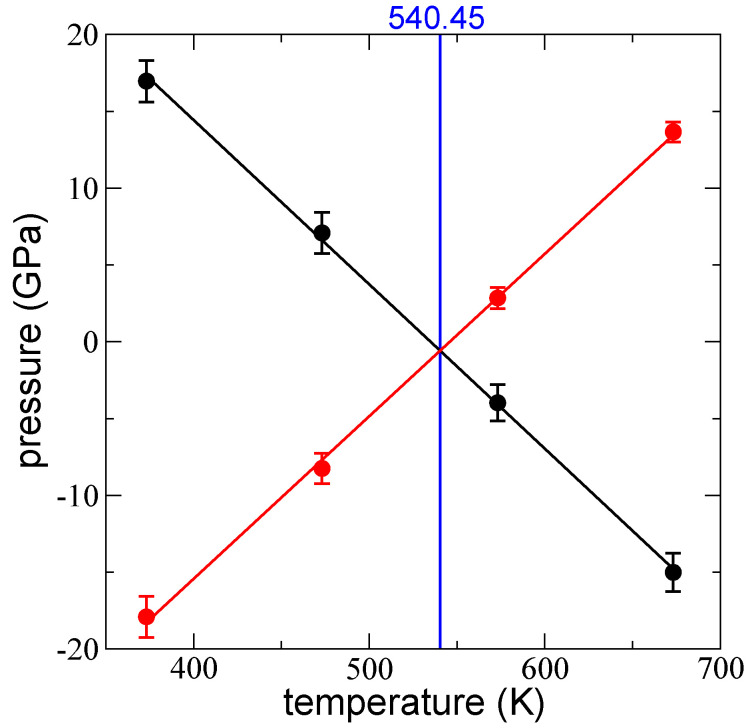
Average values of isotropic $P_{⊥,b+k}$ and $P_{∥,b+k}$ distributions (black points) in the bulk liquid region as a function of the temperature for a layer of melted polyethylene chains (C_200_) with 288 chains. Red points represent the average values of isotropic $P_{⊥,LJ}$ and $P_{∥,LJ}$ distributions (red points) in the bulk liquid region. Error bars represent the standard deviation of the distributions. The vertical blue line represents the transition temperature where the two sets changed signs.

We analyzed the transition temperature at which both the summed contribution profiles and the LJ profiles changed sign using the layer with 288 chains, which in the LJ pressure profiles developed bulk liquid regions that extended ~45 Å. The LJ pressure profiles also exhibited wide interfacial regions, characterized by the anisotropy of the pressure profiles. Figure 8 shows the average isotropic values of the pressure profiles at the bulk liquid regions for all temperatures studied. The summed pressures of kinetic and bond vibration interactions decreased linearly, while the LJ pressures increased linearly. Both datasets changed signs as they moved from systems with bulk liquid regions that are cohesive in terms of interchain interactions at lower temperatures to noncohesive systems at higher temperatures. Layers of polymeric systems dominated by noncohesive forces in the interchain interactions are expected to decompose via vaporizations or the breaking of chains at higher temperatures. LJ and the summed contributions changed sign at ~540.45 K, which was ~150 K lower than the limit of thermal degradation, which is characterized by the loss of ~5% mass at a heat rate of 1 K/min (quasi-isothermal mode) [30,31]. However, the mass loss due to vaporizations or chain breaks that end in the thermal decomposition of the layer, start at a much lower temperature. It has been experimentally determined that these changes start at an onset temperature in thermogravimetric analyses, which is the temperature at which the first mass loss is observed. In bulk HDPE, which can be considered to be a linear polyethylene, it has been established that the onset temperature is 662.15 K under a nitrogen atmosphere [53]; in contrast, the onset temperature is 596.15 K in ambient air, which compares well to our results, which were conducted under vacuum. For ultrathin films, McNeill and Mohammed [54] found that ultrathin polystyrene films with smaller thicknesses (100 Å) showed maximum temperatures of thermal stability, which are ~60 K larger than those with a thickness of 200,000 Å, therefore, the transition temperature we found for polyethylene will be lower in thicker films or bulk materials.

### 3.4. Distribution of Bond Distances

**Figure 9:**
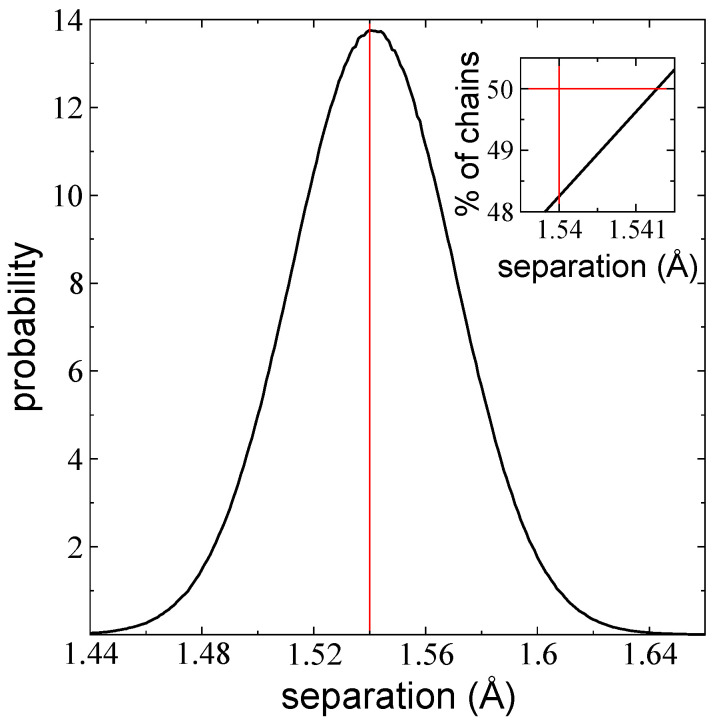
Distribution of bond separations in the bulk liquid region for a layer of melted polyethylene chains (C_200_) with 288 chains at 373.15 K. The red line represents the equilibrium value of the bond distance (1.54 Å). Inset: The percentage of chains lower than the given separation on the *x*-axis. The vertical red line represents the equilibrium separation (1.54 Å).

We verified the results for the bond vibration pressure profiles, which showed that, on average, the number of bond separations that are larger than the equilibrium value was greater than the number of bond separations that were lower than the equilibrium value. This phenomenon dominated the balance of negative forces and pressures in the pressure profiles in both the bulk liquid region and the interfacial region. Figure 9 shows the distribution of bond distances for chains in the bulk liquid region in a layer comprised of 288 chains at 373.15 K, analyzed over a period of 10 ns. It exhibits a normal distribution with an expected value that is slightly shifted to the right of the bond equilibrium value, $b_{0}$. We performed a cumulative sum starting from the lowest bond separation found in the simulation (Figure 9 inset) and found that the number of bonds lower than $b_{0}$ (1.54 Å) comprised 48.25% of the chains, while 51.75% of the bonds were higher than $b_{0}$. A total of 1.75% of the bonds at separations larger than $b_{0}$ produced the dominant negative forces in the pressure profiles. We found that the 50th percentile corresponded to a bond separation of 1.5413 Å, a value that was 0.0013 Å larger than $b_{0}$. This is a relatively small difference, but the large number of bonds in the system can result in the production and accumulation of large forces and pressures. Similar results were observed in free-standing layers of pure ethane with nanoscopic $t_{L}$ in the region of VLE [36].

### 3.5. Surface Tension

**Figure 10:**
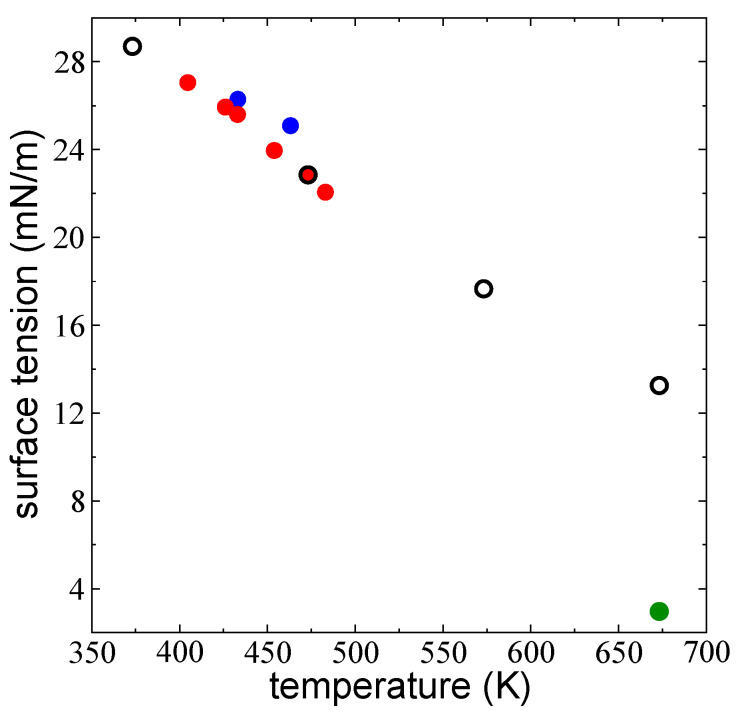
Average γ of thin liquid layers of melted polyethylene chains (C_200_) at liquid–vacuum equilibrium as a function of *T*. The black circles represent the simulation results presented in this work. The green circle represents a layer simulated at a smaller $r_{c}\;\left(2.5\;\sigma_{CH_{2}-CH_{2}}\right)$. The red circles represent the experimental results of Dee and Sauer [28] for C_150_ melted polyethylene chains at 0.1 MPa (Adapted with permission from Ref. [28]. Copyright 1992, Elsevier). Blue circles represent the experimental results of Yang et al. [55] for HDPE at 0.1 MPa (Adapted with permission from Ref. [55]. Copyright 2010, Elsevier).

To validate the pressure profiles obtained in these simulations, we calculated γ using Equation (1) for the layers with 288 chains and compared our estimates to experimental measurements [28,55]. The results for γ as a function of *T* are reported in Figure 10. Our estimations produce the expected near-linear decrease of γ with *T*, which was consistent with the experimental measurements of melted polyethylene C_150_ chains at atmospheric pressure conditions at low and moderate temperatures reported by Dee and Sauer [28]. Yang et al. [55] used longer linear chains of HDPE with $M_{w}=16.8\times 10^{4}$ and $M_{n}=0.2\times 10^{4}$ and found slightly higher values for γ in a very narrow range of temperatures. Dee and Sauer [28] studied several linear polymers with different sizes and found a relationship between γ and $M_{w}$ where larger chains exhibit greater γ. However, only small differences were observed between C_150_ and C_2000_ chains, suggesting that the observed differences between our results, which used C_200_ chains, and the experimental measurements in polymers with large $M_{w}$, are to be expected. We also studied the dependence of the measurements of γ when the simulations are carried out using a smaller $r_{c}=2.5\;\sigma_{CH_{2}-CH_{2}}$. The errors in the calculation of γ in these simulations can be compensated by the lower cost of calculation, and long-range corrections can be employed to produce better estimates of γ. Nevertheless, the dynamics of the simulations are different, and any additional phenomena studied will be biased by the low $r_{c}$ [56]. At 673.15 K, the estimated γ measure using $r_{c}=2.5\;\sigma_{CH_{2}-CH_{2}}$ is more than four times lower than the value we estimated using the full potential.

**Figure 11:**
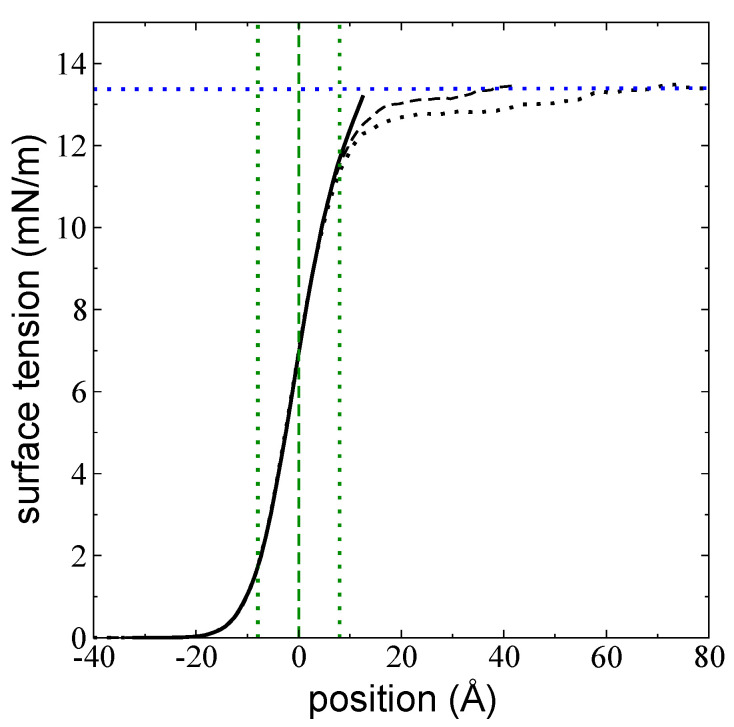
Cumulative integration profile of γ—obtained using Equation (1)—as a function of the inhomogeneous position within the simulation cell for systems of melted polyethylene chains (C_200_) at *T* = 673.15 K measured using the left interface. Continuous, dashed, and dotted black lines correspond to layers of 116 chains (the $t_{c}$), 288 chains, and 1568 chains, respectively. All profiles were displaced to align their $z_{Gds}$ to the origin of the axis in the normal direction. The dashed green line corresponds to the shifted positions of $z_{Gds}$, while the dotted green lines correspond to the shifted positions of $z_{10\%}$ (**left**) and $z_{90\%}$ (**right**). The dotted blue line shows the average value of the full magnitude of the surface tension.

The relationship between γ and $t_{L}$ was studied at 673.15 K and presented in Figure 11 using three layers with different thicknesses: $t_{L}\;$= $t_{C}$ (116 chains), 115.72 Å (288 chains), and 558.50 Å (1568 chains). Figure 11 presents the integration profile of γ as a function of the inhomogeneous position, using shifted profiles in the inhomogeneous direction to match the $z_{Gds}$ values at the beginning of the axis. We reported the profiles of a very wide layer (1568 chains) because we observed a very large interfacial region in the integration profile of γ. The three layers produced the same value for γ (integrated only over the left interface). The profile of the layer where $t_{L}=t_{C}$ ends sharply, while the intermediate layer exhibited asymptotic behavior that ended near the center of the layer (~40 Å). The widest layer also exhibited asymptotic behavior, plateauing ~62 Å from $z_{Gds}$, representing a separation of little more than twice the value of the $r_{c}$ used to calculate the interchain interactions, or four times the $t_{i}$. The profile of γ begins at positions that are well below $z_{10\%}$, and γ reached half its value at the $z_{Gds}$. The three layers exhibited the same behavior from the beginning of the integration (in the vacuum) up to $z_{90\%}$, which indicates that the outermost areas of the interfaces, which are in close contact with the vacuum, are not affected by $t_{L}$. The profiles exhibited differences beyond $z_{90\%}$, with the thinnest layers reaching the full value of γ at shorter separations from the $z_{Gds}$.

## 4. Conclusions

The interfacial and bulk pressures of free-standing layers of melted C_200_ polyethylene chains of nanoscopic thicknesses were studied between 373.15 and 673.15 K using MD simulations and the TraPPE forcefield [32]. From analyses of the $P_{*,T}$ (∗ refers to either ∥ or ⊥) and its main contributing components ($P_{*,k}$, $P_{*,LJ}$, and $P_{*,b}$), we concluded that the distribution of conformations are dominated by stretched chains that are characterized by bond distances that are greater than the equilibrium value in both the interfacial regions and the developed bulk liquid regions, which, in this study, extended to lengths of up to tens of nm. The main pressures are the negative $P_{*,b}$ due to the stretched chains and the positive $P_{*,k}$, which are one order of magnitude greater than $P_{*,LJ}$ in the bulk liquid region. The sum of the two primary contributions to $P_{*,T}$ almost cancel out $P_{*,LJ}$, which is dominated by repulsive forces at the interface (in both directions). In the developed bulk liquid regions, the character of $P_{*,LJ}$ transitioned from being attractive at lower temperatures to being repulsive at higher temperatures, with the opposite trend being observed in $P_{*,b+k}$. $P_{*,T}$ produced negative values in the bulk liquid region, which is consistent with the behavior of nanoscopic $t_{L}$ [50,51,52]. Future work will try to predict the transition thickness at which the micro- and macroscopic layers become dominated by capillary forces.

The predicted transition temperature (540.45 K) at which $P_{*,LJ}$ changes its character from cohesive to noncohesive was found to be in the vicinity of the onset temperature, which refers to the temperature at which the first changes in mass are observed via experimental thermogravimetric analyses [53]. We expect the systems simulated at temperatures larger than 540.45 K will thermally decompose in periods of time much larger than the periods of time we can simulate.

The pressure profiles produced reliable predictions for γ; the average value of γ was found to be independent of $t_{L}$, similar to results previously reported for the length of the $t_{i}$ [11], even at thicknesses as low as the limit thickness of mechanical stability. The integration profiles for γ revealed a small dependence on $t_{L}$; at larger $t_{L}$, the profile requires longer distances to reach the total value of γ. In the widest layer studied, γ only reached its maximum value ~62 Å from $z_{GDS}$, deep into the bulk liquid region.

For systems at temperatures below the predicted onset temperature of 540.45 K, the two interfacial noncohesive peaks of the $P_{⊥,LJ}$ profiles are in very close contact at thicknesses at $t_{C}$, which likely induces mechanical instabilities at $t_{L}$ < $t_{C}$. This results in the contact of noncohesive peaks without the need for large fluctuations of the film as previously reported, resulting in the generation of disjoining pressures that disrupt ultrathin films [50,51].

## Data Availability

The data presented in this study are available on request from the corresponding author.
